# Verbal Deception and the Model Statement as a Lie Detection Tool

**DOI:** 10.3389/fpsyt.2018.00492

**Published:** 2018-10-09

**Authors:** Aldert Vrij, Sharon Leal, Ronald P. Fisher

**Affiliations:** ^1^Department of Psychology, University of Portsmouth, Portsmouth, United Kingdom; ^2^Department of Psychology, Florida International University, Miami, FL, United States

**Keywords:** deception, interview, model statement, encouraging interviewees to say more, lie detection

## Abstract

We have been reliably informed by practitioners that police officers and intelligence officers across the world have started to use the Model Statement lie detection technique. In this article we introduce this technique. We describe why it works, report the empirical evidence that it works, and outline how to use it. Research examining the Model Statement only started recently and more research is required. We give suggestions for future research with the technique. The Model Statement technique is one of many recently developed verbal lie detection methods. We start this article with a short overview of the—in our view- most promising recent developments in verbal lie detection before turning our attention to the Model Statement technique.

## Verbal lie detection

DePaulo et al.'s (1) comprehensive meta-analysis of nonverbal and verbal cues to deception showed that such cues are generally weak and unreliable (2). Research has also suggested that this applies more to nonverbal cues than to verbal cues to deception: A meta-analysis about observers' ability to detect deceit when observing nonverbal and verbal cues to deception showed that when observers could only see the target person, they performed worse (52% accuracy) than when they could only hear the target person (63%) (3). This relative weakness of nonverbal cues to deceit could at least in part be explained when taking into account the strategies truth tellers and liars use when attempting to make a convincing impression on others. Truth tellers and liars employ similar strategies regarding nonverbal behavior: Both try to suppress signs of nervousness and attempt to replace them with signs that will create the impression of being honest, such as looking conversation partners into their eyes and avoiding fidgeting (scratching head, wrists etc.) (4, 5). In contrast, truth tellers and liars use different strategies regarding verbal behavior. Truth tellers are forthcoming and employ a “tell it all” strategy, whereas liars employ a “keep it simple” strategy and avoid mentioning incriminating details (6, 7). As a consequence, truthful stories often include more details than deceptive stories (1, 8).

Because researchers found nonverbal cues to be ineffective to detect deception, they refocused their efforts to focus on verbal cues. Particularly, they have tried to elicit or enhance verbal cues through specific interview techniques that exploit the different verbal strategies that truth tellers and liars employ (9). In our view, four of these efforts have shown the best results or the best potential in terms of lie detection (10, 11): (a) The Strategic Use of Evidence, (b) Assessment Criteria Indicative of Deception, (c) the Verifiability Approach, and (d) Cognitive Credibility Assessment, to which the Model Statement technique belongs. We outline these approaches briefly and refer to Vrij (10, 11) and Vrij and Fisher (12) for further details.

### Strategic use of evidence (SUE)

The aim of the SUE technique is to exploit the different strategies truth tellers and liars employ in interviews, particularly the difference in between forthcoming (truth tellers) and avoiding mentioning incriminating details (liars) (6, 13). In a SUE interview, the investigator asks questions related to the evidence s/he possesses without making the interviewee aware of possessing this evidence (i.e., asking about someone's whereabouts without revealing that CCTV footage showed that the suspect was in a shopping mall where a robbery took place). This typically leads to truth tellers' accounts being more consistent with the available evidence than liars' accounts (14). In addition, during an interview liars sometimes start to realize that the interviewer may have some evidence against them (i.e., CCTV footage about being in the shopping mall). Liars then often change their statement and provide an innocent explanation for the evidence (i.e., admitting for the first time to have been in the shopping mall, but not admitting to have been in the shop where the robbery took place). Such changes in liars' stories are called within-statements inconsistencies and liars show more of them than truth tellers (14).

### Assessment criteria indicative of deception (ACID)

The ACID interview procedure is based on the Cognitive Interview, a well-established protocol to elicit more information from cooperative witnesses through enhancing three processes: Social dynamics, memory/cognition and communication (15). In ACID, truth tellers and liars provide an initial free recall followed by instructions that stimulate communication and aid memory (16). An example of communication stimulation used in ACID is transfer of control to the respondent, and three examples of memory aids used in ACID are mental reinstatement of context, recall from another person's perspective, and reverse-order recall. ACID research has shown that, amongst other findings, truth tellers report more additional information after the initial free recall than liars (16, 17).

### Verifiability approach (VA)

The VA is based on the idea that liars face a dilemma. On the one hand, liars prefer to provide many details. This makes sense because the more details someone provides, the more likely it is that s/he will be believed (18, 19). On the other hand, liars do not wish to mention too many details. The more details they provide, the more opportunity they give to investigators to check these details and to discover their lies (19). A strategy that incorporates both seemingly conflicting goals is to provide details that cannot be verified (20). Indeed, research has shown that truth tellers typically report more details that can be checked than liars (21). Checkable details are activities that someone claims to have carried out or was witnessed by a named person, or activities that someone claimed was recorded on CCTV. In addition, activities that leave a trace (mobile phone call, text, debit/credit card purchases, and receipts) are also considered checkable. The effect that truth tellers report more checkable details than liars becomes stronger when interviewees are instructed to try to include details in their statement that the investigator can verify. Following such a request, truth tellers add more checkable details in their accounts than liars (22, 23).

### Cognitive credibility assessment (CCA)

The CCA technique comprises three elements: (i) Imposing cognitive load; (ii) Asking unexpected questions, and (iii) Encouraging interviewees to say more (24, 25).

(i) Cognitive credibility assessment: Imposing cognitive load. fMRI research has shown that in interviews lying is typically more cognitively demanding than telling the truth (26). Investigators can exploit this difference in cognitive load by making additional requests that will further increase the cognitive load truth tellers and liars experience [such as gripping an object while telling a story, (27)]. Since liars' mental resources are already depleted by the act of lying, they find it more difficult than truth tellers to cope with such additional requests (27) and the additional requests may also impair their story telling (28).

(ii) Cognitive credibility assessment: Asking unexpected questions. Liars often prepare themselves for interviews by planning answers to possible questions (7). This planning makes sense as planned answers often contain fewer cues to deceit than spontaneous answers (1). However, there is a weakness: Liars cannot know which questions will be asked. Investigators can exploit this weakness by asking a mixture of anticipated and unanticipated questions. Liars find it easier to answer the anticipated questions than the unanticipated questions, because they can give their planned answers to the former but not to the latter (29). For truth tellers, the difficulty in answering anticipated and unanticipated questions should be less pronounced. The most straightforward application of this technique is by interviewing pairs of suspects individually and comparing their answers to the expected and unexpected questions. Pairs of truth tellers showed similar overlap in their answers to expected questions as pairs of liars, but the pairs of truth tellers showed more overlap in their answers to unexpected questions than pairs of liars (30, 31). Another comparison can also be made: Comparing the overlap between expected and unexpected questions within pairs of truth tellers and within pairs of liars. Pairs of truth tellers showed a similar overlap in their answers to the expected and unexpected questions, whereas pairs of liars showed more overlap in their answers to the expected questions than in their answers to the unexpected questions (31).

(iii) Cognitive credibility assessment: Encouraging interviewees to say more. In interview settings, truth tellers typically do not provide spontaneously all the information they hold in their memory (32, 33). There are two reasons for this, a cognitive reason and a social reason.

Regarding the cognitive reason: Interviewees are unable to retrieve spontaneously all the information from their memory. Memory recall can be enhanced by using mnemonics of which asking interviewees to sketch while talking is an example (15). Sketching while narrating elicits additional information in truth tellers (34–36). Vrij et al. (37) provide four reasons for this. First, sketching is a method to mentally reinstate the context of the interviewee's experience and context reinstatement enhances memory recall. Second, sketching is a visual output which makes it more compatible with visually experienced events than the traditional oral output. Sketching facilitates recalling visual or spatial information (15), which is often the type of information interviewees discuss. Third, making a sketch is a time consuming activity. This will result in the interviewee having more time to think about the event,^1^ and this enhanced thinking may improve his/her recall of the event. Fourth, the request to sketch automatically leads to obtaining spatial information because each person/object must be positioned somewhere in the location someone sketches. Spatial information is not automatically given in a verbal response, because someone can just report who were present and which objects were present without reporting their locations (38). In the only deception experiment to date in which participants were asked to sketch while narrating (37), the difference in truth tellers reporting more additional details than liars was greater in the sketch condition than in the control condition. Truth tellers are likely to have had a richer memory of the event than liars, and truth tellers' richer memory may have led them to report more new details than liars.

The second reason why truth tellers typically do not provide spontaneously all the information they know in interview settings is a social reason: People are uncertain what and how much information they are expected to provide. The Model Statement technique addresses this social reason.

## The model statement technique

In daily life situations social rules imply that people do not report all the information they know. For example, when someone is asked by a colleague on Monday morning what s/he did during the weekend, the answer is likely to be very short: Just a few words or few sentences highlighting the main activities. Of course, interviewees will realize in formal interview settings that they need to provide more information than a few words or sentences, but they still do not know how much detail they are expected to provide. One effective way to change truth tellers' idea about how much information to provide in an interview setting is to expose them to a Model Statement, which is an example of a detailed account unrelated to the topic of the interview (39). The Model Statement works as a social comparison (40, 41) and has shown to raise the expectations amongst both truth tellers and liars about how much information they are expected to (42). A Model Statement works better than the verbal request “to provide all the details someone can remember,” perhaps because the former is a concrete example whereas the latter is an abstract instruction. It is probably easier for people to follow concrete examples than abstract instructions (43).

A Model Statement does not just elicit information, it can also be used for lie detection if certain dependent variables are analyzed. In the first two Model Statement deception studies ever published, the Model Statement facilitated the elicitation of information (39, 44). However, it did so in truth tellers and liars to a similar extent, which made the technique unsuitable for lie detection purposes when “total details” was considered as output variable. This exact pattern of results has been replicated in six out of seven ensuing studies (42, 43, 45–49), but see Porter et al. (47) as an exception. In other words, the Model Statement technique elicits more information in both truth tellers and liars, but cannot distinguish between truth tellers and liars based on the total amount of information.

For the Model Statement technique to work as a lie detection tool it is important to consider the quality rather than the quantity of information that is reported. The first Model Statement deception study (39) already hinted at this: Although truth tellers and liars provided a similar amount of information after exposure to a Model Statement, the information provided by truth tellers sounded more plausible than that of liars. That the quality of details rather than the quantity of details distinguish truth tellers from liars makes sense. Both truth tellers and liars realize after exposure to a Model Statement that they are expected to provide many details (42). The amount of details is thus unlikely to distinguish between the two groups. The type of detail becomes relevant because it takes into account the different cognitive abilities of truth tellers and liars and the different strategies they use to appear convincing.

Studies to date gave insight into two types of detail that could distinguish truth tellers from liars after exposure to a Model Statement, the number of complications (37, 49) and the number of peripheral details (43) that were reported. A complication is “an occurrence that makes a situation more difficult than necessary” (37). Examples of complications are “The sailing race was canceled, because there was not enough wind” and “When we arrived at the museum it was closed”; “Initially we did not see our friend, as he was waiting at a different entrance”) (37, 49). Complications occur more often in truthful statements than in deceptive statements (8, 50). In interviews, liars prefer to keep their stories simple (7), but adding complications makes the story more complex. A Model Statement increases the number of complications interviewees report, particularly in truth tellers (37, 49). Complications are often not about key aspects of the activities that someone describes, and the story can be well understood without reporting the complications. Take for example, when someone describes traveling to a holiday destination. All sorts of complications that happen en route to a holiday destination are not necessary to understand the travel to the holiday destination (someone forgot to bring a valuable item; taxi turned up late; traffic on the road; airplane delayed; late gate change at the airport). Therefore, truth tellers may leave at least some of them out when they are not exposed to a Model Statement. Liars are reluctant to provide complications in order to keep their story simple. As a result, truth tellers are more likely than liars to report more complications after being exposed to a Model Statement.

A second measure that takes truth tellers' and liars' different strategies into account is distinguishing between core or peripheral details (43). Core details are details that, if changed, can result in changes in the basic and most important part of the story; details that have no such impact are considered peripheral (51). Thus, if someone describes attending an Adele pop concert, all details about the actual concert are core details whereas information about drinks in the pub before and after the concert, are peripheral details. Both truth tellers and liars realize that they need to provide more details after exposure to a Model Statement (49). Truth tellers, who have actually experienced an event (e.g., attending an Adele pop concert), will be able to provide more core and peripheral information, by employing a “tell it all strategy” (7). For liars, who have not experienced an event (e.g., did not attend an Adele concert), providing core information is more difficult and risky. It is difficult because they have to make up information and it is risky because the information may provide leads to investigators that they can check. Thus, liars may avoid providing too many core details in an attempt to minimize the risk of presenting incriminating information (6, 21), but may compensate this by providing peripheral details in an attempt to provide a sufficient amount of detail. In the only Model Statement deception experiment distinguishing between core and peripheral details to date, the latter assumption was supported: In the Model Statement present condition liars reported more peripheral details than truth tellers, whereas no difference in peripheral details emerged in the control condition (43).

## How to use a model statement in real life

We believe that the Model Statement technique should be used as a within-subjects technique, as employed by Leal et al. (43). Thus, first the interviewee should be invited to initially report via an open-ended question all s/he can remember about the event under investigation. This should then be followed by a Model Statement after which the interviewee should again be invited to report via an open-ended question all s/he can remember, but this time by taking into account the amount of detail s/he heard in the Model Statement. Investigators should then listen to the number of new complications reported in the second recall and the amount of new peripheral information reported in the second recall.

### Three practical elements merit attention

First, use a within-subjects structure when applying the Model Statement technique. Within-subjects comparisons are better for lie detection purposes than between-subjects comparisons (52). In a between-subjects comparison, the interviewee would be asked to report the event only once and to do this after exposure to the Model Statement. The amount of information an interviewee provides depends on many factors, including his/her personality [some people talk more than others (53–55), the situation (some events are richer in detail than others) or preparedness for the interview [pre-planned answers often contain more words than spontaneous answers, e.g. (56)]. In a within-subjects comparison, it does not matter how detailed an initial answer is or how many complications someone initially provides (which is largely influenced by personality, situation and preparedness). The only relevant measure is the number of peripheral details and complications that are *added* (more likely to be influenced by veracity).

Second, the Model Statement should be unrelated to the topic of investigation so that it does not give liars the chance to “copy” the example and use it in their own statement. In our research, we use a 734 words Model Statement in which a young man describes his experiences when attending a Formula 2 motor race, commencing where the drivers go to their grid position prior to the start of the race. This is an atypical event that does not give interviewees the opportunity to copy details.

Third, our Model Statement is an authentic experience (the person really attended a Formula 2 motor race), which we think is important. True experiences sound more realistic than made-up experiences and are therefore more powerful. It becomes even worse when someone fabricates a model statement on the spot. It typically is not detailed enough and often sounds what it actually is: a made-up story. We always present the Model Statement in the format of an audiotape. However, other ways to present the Model Statement are possible. We return to this point in the next section.

## Future research

Unfortunately, many lie detection techniques are taught to practitioners without solid empirical evidence to back them up, which we consider a particularly poor and potentially harmful practice (12, 57). Many research avenues for Model Statement deception research are possible. We will conclude this article by discussing five more research ideas in somewhat more detail.

First, an obvious but important research endeavor would be replication of studies that have been carried out so far, ideally by different groups of researchers in different labs. Most Model Statement research to date comes from Vrij's lab but much stronger conclusions could be drawn if Vrij's lab findings are replicated in other labs. This refers in particular to research related to complications and core/peripheral details, as research in that area is still scarce. At the same time, those researchers could then search for other variables than complications or peripheral details on which truth tellers and liars may differ after exposure to a Model Statement.

Second, research should be carried out manipulating the content of the Model Statement. Will it have an effect on interviewees' recall? People experience activities through their perceptual senses: They see, hear, touch, smell, or taste things. An event interviewees are asked to describe may contain more information about some of these perceptual sources than about others. Will it help or hinder lie detection if interviewees are exposed to a Model Statement that corresponds with their perceptual experience? For example, if the experience the interviewee talks about contains many auditory experiences, will it then be beneficial to use a Model Statement that focuses on auditory experiences? On the one hand it may help truth tellers to recall more details they have experienced through the particular sense(s) emphasized in the Model Statement but, on the other hand, it may give liars an idea what type of information to fabricate.

Third, thus far we have always used an audiotaped Model Statement. This could be played via a loudspeaker but also from a mobile phone. Alternatives are that the investigator reads out an example or that interviewees read a written text of a Model Statement. Until tested it is unclear which—if any—modality works best for discriminating between truth tellers and liars.

Fourth, from training we give in the Model Statement technique (58), we know that interviewees quickly understand that they are requested to provide more details than they initially thought they had to provide. This may result in different mental processes in truth tellers and liars. Adding information should be easier for truth tellers than for liars, as truth tellers can go back to their memory, whereas liars have to think what made-up details to add to their stories. Consequently, liars may listen less to the content of the Model Statement than truth tellers, because liars cannot listen to the Model Statement and think of details to add at the same time. If so, truth tellers and liars might be able to report back the content of the initial part of the Model Statement to an equal extent as at the initial stage both are listening to the Model Statement. However, after this stage, liars should switch off and start thinking about the details they will add. From this point onwards, we expect liars to report back less of the content of the Model Statement than truth tellers.

Fifth, a meta-analysis summarizing Cognitive Interview research showed that “report everything” instructions result in interviewees reporting more information without a reduction in accuracy (59). We expect a Model Statement also to have this effect on truth tellers—more information without a reduction in accuracy—but believe that this issue is important enough to be examined empirically.

## Author contributions

AV wrote the initial article and received comments from SL and RF.

### Conflict of interest statement

The authors declare that the research was conducted in the absence of any commercial or financial relationships that could be construed as a potential conflict of interest.

## Footnotes

^1^Slowing down the output process also makes it easier for the interviewer to understand the interviewee's statement, which probably enhances the communication between interviewer and interviewee.
